# Cerebral edema in intracerebral hemorrhage: pathogenesis, natural history, and potential treatments from translation to clinical trials

**DOI:** 10.3389/fstro.2023.1256664

**Published:** 2023-09-29

**Authors:** Kailash Krishnan, Paula Bermell Campos, Thanh N. Nguyen, Chia Wei Tan, Siang Liang Chan, Jason P. Appleton, ZheKang Law, Milo Hollingworth, Matthew A. Kirkman, Timothy J. England, Christine Roffe, Mary Joan Macleod, Jesse Dawson, Ulvi Bayraktutan, David J. Werring, Nikola Sprigg, Philip M. Bath

**Affiliations:** ^1^Stroke, Department of Acute Medicine, Nottingham University Hospitals NHS Trust, Nottingham, United Kingdom; ^2^Stroke, Academic Unit of Mental Health and Neuroscience, University of Nottingham, Nottingham, United Kingdom; ^3^Department of Neurology, Boston Medical Centre, Boston University Chobanian and Avedisian School of Medicine, Boston, MA, United States; ^4^Department of Stroke, University Hospitals Birmingham NHS Foundation Trust, Birmingham, United Kingdom; ^5^College of Dental and Medical Sciences, Institute of Applied Health Research, University of Birmingham, Birmingham, United Kingdom; ^6^Department of Medicine, Faculty of Medicine, National University of Malaysia, Kuala Lumpur, Malaysia; ^7^Department of Neurosurgery, Nottingham University Hospitals NHS Trust, Nottingham, United Kingdom; ^8^Department of Stroke, Derby Royal NHS Foundation Trust, Derby, United Kingdom; ^9^Stroke Research in Stoke, Institute of Science and Technology, Keele University, Stoke-on-Trent, United Kingdom; ^10^Department of Clinical Pharmacology, School of Medicine, Medical Sciences and Nutrition, University of Aberdeen, Aberdeen, United Kingdom; ^11^Institute of Cardiovascular and Medical Sciences, University of Glasgow, Glasgow, United Kingdom; ^12^Department of Brain Repair and Rehabilitation, UCL Stroke Research Centre, London, United Kingdom; ^13^UCL Institute of Neurology, National Hospital for Neurology, National Hospital for Neurology and Neurosurgery, London, United Kingdom

**Keywords:** intracerebral hemorrhage, cerebral edema, translation, clinical trial, treatment, pathophysiology

## Abstract

Acute intracerebral hemorrhage is the most devastating stroke subtype and is associated with significant morbidity and mortality. Poor prognosis is associated with primary brain injury from the presenting hematoma, and despite advances in clinical trials of evacuation or reducing expansion, management is largely limited to supportive care and secondary prevention. Recent research has led to a better understanding of the pathophysiology of the cerebral edema surrounding the hematoma (perihematomal edema) and the identification of treatment targets and potential interventions. Some therapies have progressed to testing in phase 2 and 3 clinical trials, while novel agents are in development. This review focuses on the pathogenesis of perihematomal edema and its natural history and summarizes the results of potential interventions including preclinical and clinical studies. This review also lists the gaps in the current knowledge and suggests directions for future trials of perihematomal edema that could potentially change clinical practice.

## Introduction

Spontaneous intracerebral hemorrhage (ICH) accounts for approximately 20% of all strokes and affects about 3 million patients worldwide each year (GBD 2019 Stroke Collaborators, 2019). ICH is devastating, with a mortality of ~40% at 1 month, and more than two-thirds of survivors remain dependent, requiring long-term care (van Asch et al., 2010; Krishnamurthy et al., 2020). Compared to advances in the management of acute ischemic stroke, the treatment for ICH lags significantly. Poor prognosis is associated with the hematoma size, location, and intraventricular hemorrhage. Hence, randomized clinical trials have assessed limiting the hemorrhage size and expansion through hemostasis, lowering blood pressure (BP), and surgery (Broderick et al., 1993; Mendelow et al., 2005, 2013; Anderson et al., 2008; Investigators, 2015; NCT02880878, 2023). Except for surgical evacuation of a hematoma in a highly selected group of patients (Ratcliff et al., 2023), no other intervention has been shown to improve functional outcomes. Therefore, the management of ICH remains largely supportive. Survivors of ICH are at risk of death or severe disability due to the cerebral edema that surrounds the hematoma (perihematomal edema [PHE]) (Venkatasubramanian et al., 2011; Balami and Buchan, 2012; Yang J. et al., 2015; Hurford et al., 2019). In a large-volume ICH, the accompanying swelling can increase the mass effect and lead to brain herniation (Zazulia et al., 1999). Hence, PHE is emerging as a key component of secondary brain injury and a potential surrogate outcome measure for preclinical and clinical trials (Venkatasubramanian et al., 2011; Yang Z. et al., 2015). Understanding the mechanisms and natural history of PHE is therefore important, and the development of potential therapeutic agents is of interest to clinical trialists and researchers.

**Figure 1:**
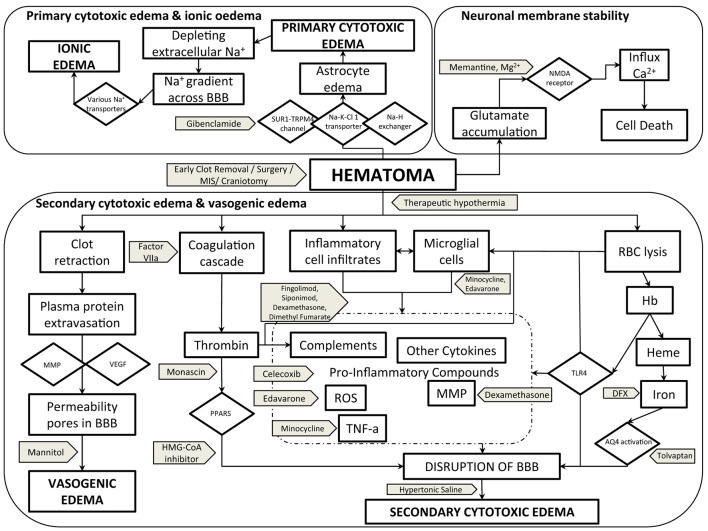
Pathogenesis of PHE cerebral edema with potential therapeutic targets and interventions. AQ4, aquaporin-4; BBB, blood-brain barrier; Ca^2+^, calcium ion; DFX, deferoxamine; Hb, hemoglobin; Heme, HMG-CoA-3-hydroxy-3-methylglutaryl-coenzyme A; MIS, minimal invasive surgery; MMP, matrix metalloproteinase; Mg^2+^, magnesium ion; Na^+^, sodium ion; Na-H exchanger, sodium–hydrogen exchanger; Na-K-Cl-1 transporter, sodium–potassium–chloride-1 transporter; NMDA, N-methyl-D-aspartate; PPAR, peroxisome profilerator-activated receptor; RBC, red blood cells; ROS, reactive oxygen species; SUR1-TRPM4 channel, sulfonylurea receptor-1–transient receptor potential melastatin 4 channel; TNF-α, tissue necrosis factor–alpha; TLR4, toll-like receptor-4; VEGF, vascular endothelial growth factor.

The pathogenesis of PHE and accompanying brain injury are not yet fully understood but include vasogenic, cytotoxic, inflammatory, and oxidative mechanisms accompanied by disruption of the blood-brain barrier (BBB) (Figure 1). In this narrative review, we focus on these mechanisms, the natural history of PHE, neuroimaging measures, and laboratory parameters that may be associated with the pathogenesis, and summarize potential interventions, including data from translational and clinical studies. We also list the gaps in the current knowledge and suggest directions for future trials of cerebral edema that could potentially change clinical practice.

## Natural history and pathogenesis of cerebral edema

The precise natural history of PHE is unclear, but it is thought to evolve over three phases.

### The hyperacute phase

This phase of edema begins within the first hours of ictus, with the mechanism being vasogenic from activation of the coagulation cascade, leading to a retraction of the presenting hematoma (Xi et al., 1998), a decrease in hydrostatic pressure in the PHE region, and an exudation of serum plasma proteins (Reulen et al., 1977). The oncotic pressure gradient generated by the exudated proteins leads to increased brain swelling from the movement of water into the perihematomal space (Wagner et al., 1996). Such changes can be identified visually in the first few hours of stroke on a plain computed tomography (CT) or magnetic resonance imaging (MRI) scan as an area of low attenuation or high T2 or fluid attenuation inversion recovery signal surrounding the hematoma, respectively (Enzmann et al., 1981; Linfante et al., 1999). Preclinical studies have shown that the BBB is intact for the first few hours during this period and that permeability gradually increases (Yang et al., 1994; Xi et al., 1998). Therefore, a change in PHE volume during this phase is likely to be mediated by hematoma contraction and the pressure gradients generated by plasma proteins (Wagner et al., 1996).

As retraction of the hematoma continues, there is a further increase in PHE. Large swelling could increase intracranial pressure and decrease blood flow to the perihematomal region if the collateral circulation is not recruited and able to compensate. Research has shown that the perihematomal region is in a state of reduced metabolism (“hibernation”) and that autoregulation is preserved in the first few hours after ictus (Kim-Han et al., 2006; Ironside et al., 2019).

In addition to vasogenic edema, there is cellular/cytotoxic swelling because of a failure of the energy-dependent ion channel sodium-potassium adenosine triphosphatase (Na+K+ATPase) (Kahle et al., 2009). Na+K+ATPase is responsible for maintaining the transmembrane electrochemical balance and failure or dysfunction leads to excess influx of intracellular sodium and water (Kahle et al., 2009). Studies indicate that the sulfonyl-receptor-1–transient receptor potential melastatin 4 (SUR1-TRPM4) channel is also involved (Simard et al., 2006). Once activated, SUR1-TRPM4 not only leads to water accumulation and ionic dysfunction but also mediates BBB damage and the lining of endothelial cells (Simard et al., 2006, 2009). As cytotoxic edema continues, more water enters the brain through aquaporin-4 channels (AQP4), which are located on astrocytes (Qing et al., 2009). The activation of AQP4 induces water influx to compensate for the ionic imbalance from failure of Na+K+ATPase (Pollay et al., 1983). Studies suggest that the opening of AQP4 is linked to SUR1-TRPM4 (Stokum et al., 2018).

Cytotoxic edema is also potentiated through activation of the Na+-K+Cl cotransporter, which is a membrane protein on brain endothelial cells and excess extracellular glutamate after stroke (Shuaib et al., 2003). Glutamate is an important neurotransmitter and is normally released into the neuronal synaptic cleft (Collingridge et al., 2013). Its reuptake occurs at the presynaptic terminals and adjacent glial cells. In acute ICH, excess glutamate binds to N-methyl D aspartate (NMDA) receptors on postsynaptic neurons, which, in turn, facilitates the uncontrolled entry of calcium, causing apoptosis or death (Zheng et al., 2016; Ironside et al., 2019). Furthermore, glutamate can bind to other receptors facilitating an influx of sodium and water (Kritis et al., 2015).

It is important to highlight that vasogenic and cytotoxic edema are not exclusive and that the mechanisms are interlinked (Ironside et al., 2019).

### The intermediate phase

The next phase of PHE continues over the next 24–48 h of ictus when the volume has been shown to increase and could reach the maximum (Olivot et al., 2010; Parry-Jones et al., 2015; Zheng et al., 2016). Studies have reported that this time window could be longer and the variation could be explained in the timing of follow-up scans and measures used to assess edema (Arima et al., 2009; Venkatasubramanian et al., 2011; Parry-Jones et al., 2015). Observational data indicate that edema in lobar ICH can increase quickly in the first few days and reach a larger volume than deep ICH (Gebel et al., 2002; Grunwald et al., 2016; Wu T. Y. et al., 2017). This could be because lobar regions in the brain have more space and fewer adjacent tightly packed white matter tracts compared to deep ICH, so there is less resistance to extending the swelling (Grunwald et al., 2016).

Pathophysiologically, the intermediate phase is characterized by events including the breakdown of the hematoma, the activation of thrombin, inflammation, and microglial/macrophage activation (Xi et al., 2003; Zhao et al., 2007a, 2015a; Keep et al., 2012). This results in damage to the BBB, which, in turn, increases inflammation by promoting leukocyte infiltration, ionic imbalance, and the entry of toxic substances into the brain interstitium. Studies have shown that the increased permeability of the BBB is facilitated by cytotoxic edema and ionic failure that occurred in the hyperacute phase (Reulen et al., 1977; Kahle et al., 2009). There are a number of molecules and signaling pathways involved in the intermediary phase, and the key components are discussed.

## Thrombin

The immediate response to intracerebral hemorrhage is activation of the coagulation cascade to limit bleeding and here, thrombin has an important role. However, experimental studies have shown that in high concentrations as in acute stroke, thrombin induces edema independent of fibrinogen and causes the release of nitric oxide and cytokines, including tumor necrosis factor–alpha (TNF-α), interleukin (IL)-12, and IL-6 (Xi et al., 1998, 2003). In addition, thrombin promotes recruitment and infiltration of neutrophils, lymphocytes, and macrophages into the perihematomal region by inducing chemotactic substances and adhesion molecules (Xi et al., 2003). Thrombin also directly stimulates microglia through specific proteinase-activated receptors (PARs) (Chen et al., 2022).

The activation of thrombin can cause an opening of the BBB through PARs and damage the basement membrane by activating metalloproteinases (MMPs) (Chen et al., 2022). MMPs are proteolytic enzymes, which degrade components of the basement membrane, including collagen, gelatin, laminin, and fibronectin. Thrombin can also trigger activation of Src kinase (a proto-oncogene protein with kinase activity), which, in turn, potentiates damage to astrocytes and endothelial cells, the main cellular component of the BBB (Xi et al., 2003).

## Neuroinflammation

In response to thrombin activation and damage to the BBB, leukocytes enter the perihematomal region. Studies have shown that the activation of a specific receptor, toll-like receptor 4 (TLR-4), on the surface of neutrophils and macrophages is important for infiltration (Yang J. et al., 2015). The activation of TLR-4 begins within hours after a stroke and continues for about a week (Yang J. et al., 2015). By inducing the expression of a transcription factor, nuclear factor–kappa B (NF-κB), on microglia and macrophages, TLR-4 signaling also stimulates the release of pro-inflammatory cytokines (e.g., IL-1, IL-6, TNF-α, and IL-β), signaling pathways generating free radicals, and glutamate (Sansing et al., 2007; Yang J. et al., 2015).

Neutrophils are the earliest to reach the perihematomal region, and studies suggest that the peak activity is at ~72 h. Neutrophils seem to contribute to cerebral edema by generating reactive oxygen species (ROS) and pro-inflammatory protease enzymes that damage the BBB (Wang and Dore, 2007). The activation of microglia occurs later, and studies have shown this to continue for ~2 weeks (Zheng et al., 2020). The inflammation mediated by microglia is driven by its M1 phenotype through the release of cytokines, chemokines, ROS, and nitric oxide (Wang and Dore, 2007). The activity of the M1 phenotype is maintained by activated astrocytes and lymphocytes (T1 helper cells) (Tschoe et al., 2020). At ~1 week of ictus, the M1 phenotype converts to the M2 phenotype, and this begins the process of resolution of inflammation, scar formation, and brain repair (Wu J. et al., 2017). The conversion from the M1 to the M2 phenotype is potentiated by anti-inflammatory cytokines (IL-4, IL-10, IL-13), lymphocyte T2 helper cells and a number of transcription factors (Chhor et al., 2013). Hence, the effects of microglia after ICH are either pro-inflammatory or neuroprotective and time-dependent. The anti-inflammatory effect of the M2 phenotype has led to testing of potential treatments, which is discussed later.

The disruption to the BBB also leads to the activation of the complement system in the perihematomal region (Lee et al., 1995). Both complement C3a and C5a are anaphylatoxins and induce endothelial cells and infiltration of pro-inflammatory cells (Lee et al., 1995; Holste et al., 2021). Complement activation also leads to the formation of the membrane attack complex (MAC), which causes erythrolysis. MAC is known to be directly toxic to neurons, glial cells, astrocytes, and the BBB (Holste et al., 2021).

## The late phase

Although erythrolysis begins early after ICH, the late phase of edema is mainly medicated by the release of toxic products, including hemoglobin and iron, and phagocytosis (Ironside et al., 2019).

Hemoglobin has been shown to inhibit the enzyme Na+K+ATPase and induce the release of ROS and peroxidation of membrane lipids, leading to neuronal death (Bautista et al., 2021). Haem has been shown to activate microglia through TLR-4, and this, in turn, can induce NF-κB through a complex system of gene expression or signaling pathways (Tschoe et al., 2020). This process leads to more release of inflammatory cytokines and brain injury. (Tschoe et al., 2020).

Following the breakdown of the hematoma, iron is first observed in the perihematomal region at day 1, reaching peak level at approximately 7 days, and can continue to remain at that level for approximately 2 weeks (Qing et al., 2009). There is significant evidence that excess iron leads to brain damage (Xi et al., 2002). One mechanism is through the release of toxic free radicals (“ferroptosis”) (Bautista et al., 2021). Experimental studies have also shown that iron induces inflammation by generating ROS and MMP-9 and BBB dysfunction and increases cytotoxic swelling by activating AQP4 (Qing et al., 2009).

Phagocytosis leads to brain repair and is mediated by the infiltrated microglia and macrophages in the perihematomal region (Galloway et al., 2019). Phagocytosis includes the following steps: anchoring, internalization, and, finally, processing of the products of hematoma breakdown (Hochreiter-Hufford and Ravichandran, 2013). The process itself is complex and regulated by multiple receptors and enzymes that are present on the surface of the microglia and macrophage itself (Hochreiter-Hufford and Ravichandran, 2013; Galloway et al., 2019). As phagocytosis continues, ROS and toxic free radicals are generated, which can lead to more neurotoxicity (Hu et al., 2016). Preclinical studies have shown that accelerating phagocytosis could limit the period of exposure of the brain to toxic products and lead to recovery (Zhao et al., 2007a, 2015a).

## Hormones, peptides, and toxic free radicals

It is suggested that, after ICH, excess levels of the antidiuretic hormone vasopressin induce edema and inflammation by activating astrocytes (Hertz et al., 2014). Evidence also indicates that vasopressin increases BBB permeability and water accumulation through the expression of AQP4 (Hertz et al., 2014; Zhao et al., 2015b).

Recent work has shown that endothelin-1, a hormone that is released by endothelial cells, increases within hours after stroke and correlates with BBB hyperpermeability (Li et al., 2022). In addition, endothelin-1 is linked to genes that regulate the secretion of cytokines, inflammation, the release of toxic free radicals, and iron metabolism (Wang et al., 2013).

An *in vivo* microdialysis study showed that apart from glutamate, there is an excessive extracellular accumulation of amino acids, including taurine, glycine, and asparagine in acute ICH (Shuaib et al., 2003). The role of these amino acids in the pathogenesis of inflammation and prognosis is unclear and needs further exploration (Kanthan et al., 1995; Shuaib et al., 2003).

Following the third phase of cerebral edema, some studies suggest a gradual decline in volume while others suggest otherwise. One study found that edema volume at 1 month was similar to that observed early after ictus (Fung et al., 2016). Peng et al. showed that edema volume at 2–3 weeks was much higher than baseline and was associated with poor outcomes (Peng et al., 2019). Another observational study reported that PHE can last up to 2 months (Chen et al., 2021).

## Blood pressure

BP is elevated in ~70% of patients with acute stroke and is associated with poor outcomes (Leonardi-Bee et al., 2002). Following the loss of cerebral autoregulation, high BP in ICH is associated with increased rebleeding, hematoma expansion, and PHE (Willmot et al., 2004). A history of high BP is also relevant as shown by analysis from the Intensive Blood Pressure Reduction in Acute Cerebral Hemorrhage (INTERACT) trial, which showed a significant association with an increase in edema at 72 h compared to baseline (Arima et al., 2009), In addition, BP variability is linked to the pathogenesis of edema in the hyperacute and subacute phases, but the mechanisms are not fully understood (Sykora et al., 2009; Havenon et al., 2018). One explanation is that the variation in BP directly increases the hydrostatic pressure and oncotic pressure in the PHE region (Sykora et al., 2009). Another explanation is that significant fluctuation in BP induces the release of pro-inflammatory cytokines, hyperglycemia, BBB dysfunction, and vasogenic edema (Sykora et al., 2009).

To date, no studies have prospectively tested the effects of BP lowering on PHE, but *post-hoc* analyses of two trials, INTERACT-2 and the Antihypertensive Treatment in Acute intracerebral hemorrhage (ATACH-2) trial, suggest that the rate of edema expansion was less in participants randomized to intensive treatment (Yang J. et al., 2015; Leasure et al., 2019). It is noteworthy that the majority of participants in INTERACT-2 and ATACH-2 had deep ICH, and one reason for the observed result could be that there is a difference in hematoma dynamics when compared to other brain locations (Seiffge et al., 2022). The trials excluded patients presenting in later-time windows, with large ICH volume and very high systolic BP (>200 mm Hg), and so the pathogenesis of PHE in such patients needs further research (Anderson et al., 2013; Bath et al., 2019).

## Neuroimaging measures of PHE

Studies have used various measures of PHE to assess the effect on prognosis. Some studies have used absolute PHE volume in the first hours after stroke and others have suggested the change from baseline to be useful (Sansing et al., 2003; Appelboom et al., 2013; Yang J. et al., 2015). Some authors have postulated using the ratio of PHE per mL of ICH size/volume (relative edema), but it can be difficult to estimate the effects if the hematoma itself is large (Staykov et al., 2011; Appelboom et al., 2013).

Recent work has proposed that the absolute or relative edema change per hour could be more useful as it reflects the actual speed at which the pathogenesis occurs, but this needs to be tested in larger studies (Grunwald et al., 2016).

It may be that examining the peak/highest value of PHE is more appropriate, but performing sequential scans in unwell ICH patients may not be practical (Staykov et al., 2011; Venkatasubramanian et al., 2011). However, the change in PHE is dependent on the hematocrit, which is the proportion of blood volume occupying an erythrocyte, and is known to be higher in males (Venkatasubramanian et al., 2011). A higher hematocrit could therefore expose the brain to more products of erythrolysis over time, and this could delay the peaking of edema. By comparison, in intraventricular hemorrhage, the peak value of edema can be reached more quickly as the hematocrit is diluted by the volume of cerebrospinal fluid (Venkatasubramanian et al., 2011).

Because the development of PHE is linked to ICH volume, it is difficult to assess the independent effects in the first few hours of stroke. Parry-Jones et al. suggested that edema extension distance (EED) or measuring the change in OED over time might be able to overcome this issue (Parry-Jones et al., 2015). OED is the linear measure of the extension of swelling from the boundary of the hematoma, and so theoretically, it should be independent of the hematoma. However, OED is based on the assumption that a hematoma is elliptical in shape and so may not be applicable to irregular ICH (Parry-Jones et al., 2015).

## Laboratory testing for PHE

Studies have shown that high serum glucose at baseline is linked with the pathogenesis of PHE (Esposito et al., 2002; Qureshi et al., 2011). Hyperglycemia has been shown to mediate this effect through the release of TNF-α and IL-1 (Esposito et al., 2002). Other laboratory measures, including a high red blood cell count, increased platelet count, and increased hematocrit, are associated with an increase in PHE in the first week after symptom onset (Sansing et al., 2003; Venkatasubramanian et al., 2011). It is suggested that an increase in thromboplastin time correlates with the expansion of PHE, which implies the activation of the coagulation system (Sansing et al., 2003). A meta-analysis including 2,176 patients analyzed the prognostic effect of neutrophils and lymphocytes on outcomes after ICH (Liu et al., 2019). The results showed that a high neutrophil-to-lymphocyte ratio predicted an increased risk of early death, death, or disability at day 90 (Liu et al., 2019). Pathophysiologically, this can be explained as ongoing inflammation in a patient with a weakened immune system who is likely to have a poor outcome.

## Prognostic effects of PHE

With advances in understanding the natural history of PHE, there is interest in assessing the prognostic effects. The mechanisms are not yet understood, and one reason is because the pathogenesis of PHE is linked to the presenting hematoma, which could confound the clinical effects (Gebel et al., 2002; Arima et al., 2009; Wu J. et al., 2017). In patients with ICH volume <30 cm^3^, observational data have shown that the presence of PHE at baseline increases the risk of death or disability at day 90 (Appelboom et al., 2013; Murthy et al., 2015). One explanation is that in small hematomas, a modest increase in the mass effect from PHE could lead to clinical deterioration. However, such patients could be potentially eligible for testing interventions for edema, which, in turn, could improve outcomes.

After adjusting for ICH volume, studies have used other measures of PHE and assessed the effect on clinical outcomes. Gebel et al. reported that there was an inverse correlation between relative PHE and death or disability at day 90 but other authors found no association (Gebel et al., 2002; Arima et al., 2009; Staykov et al., 2011; Volbers et al., 2016). Another study examined EED and found that a large deviation induced by swelling increased the risk of midline shift and brain herniation (Wu J. et al., 2017). Recently, Urday et al. showed that the peak increase in PHE between baseline and 24 h was independently associated with death at day 90 (Urday et al., 2016). Studies have also attempted to assess the effects of PHE beyond the hyperacute phase and shown that an increase up to 72 h predicts poor functional outcomes (Sansing et al., 2011a; Li et al., 2013; Bakhshayesh et al., 2014; Yang J. et al., 2015; Urday et al., 2016).

Research suggests that the prognostic effects of PHE depend on the location of the stroke itself (Appelboom et al., 2013; Grunwald et al., 2016; Leasure et al., 2019). This could be highly relevant in deep hematomas in the thalamus or basal ganglia, as these structures are important for maintaining consciousness, movement, and executive function. It is therefore intuitive that PHE or an increase in volume in these regions could worsen damage and result in poor outcomes (Grunwald et al., 2016; Leasure et al., 2019).

It is important to highlight that the aforementioned studies have been retrospective; varied in inclusion criteria, the modality of neuroimaging, the timing of scanning, and outcome measures; and utilized heterogeneous measures of PHE. Furthermore, little is reported on the late phase of edema and how this affects outcomes after ICH. More research is needed to understand these temporal relationships in prospective studies, including a large number of patients.

## Potential interventions for cytotoxic edema

### Targeting SUR1-TRPM4 channels

Glyburide, also known as glibenclamide, a sulfonylurea receptor (SUR1) antagonist and potent inhibitor of TRPM4 channel in the brain, has emerged as a potential treatment for cerebral edema (Simard et al., 2006). In acute stroke, the low pH in the brain resulting from the lack of energy and hypoxia facilitates the entry of glibenclamide through the BBB (Simard et al., 2006). In one preclinical model, glibenclamide reduced PHE, protected the BBB, and reduced the expression of MMP. In addition, treatment was associated with improved neurological function (Zhou et al., 2018). However, Kung et al. reported that glibenclamide had no effect, but this could be explained by the different ICH model that was used (Kung et al., 2021) (Table 1). In a small clinical trial, treatment using a patented form of intravenous glibenclamide in ischemic stroke was associated with a reduction in the midline shift and MMP-9 levels measured at 24–72 h compared to controls (Sheth et al., 2016). Based on these results, a recent phase 2 trial tested the safety and efficacy of oral glibenclamide in basal ganglia ICH-associated cerebral edema within 72 h of symptom onset (NCT03741530, 2022). The results showed that treatment significantly reduced the proportion of poor outcomes at day 90, but glibenclamide was associated with a higher incidence of hypoglycemia, although this was asymptomatic (Zhao et al., 2022).

**Table 1:** Summary of key preclinical studies testing interventions for cerebral edema in intracerebral hemorrhage.

**Agent**	**References**	**Method**	**Findings**
*Osmotic agents*			
Mannitol and hypertonic saline	Qureshi et al. (1999)	•Canine—Autologous blood model •IV mannitol 1 gm/kg vs. 3% NaCl 5.3 ml/kg vs. 23.4% NaCl 0.7 ml/kg •2 h after induction	•Reduction in ICP after administration of NaCl (3% and 23.4%), comparable with isomolar mannitol •Rebound increased in ICP on mannitol and 23.4% NaCl •Water content was lowest in most regions in the 3% NaCl group and highest for the mannitol group
	Schreibman et al. (2018)	•Rat—collagenase model •2 groups: moderate and severe ICH •Mannitol 20% 1g/kg vs. HTS 23,4% 0.7ml/kg vs. no treatment •5 h after ICH and every 12 h up to 4 doses	•Reduced hemispheric swelling and mortality at 48 hours •Reduced inflammation
*Anti-inflammatory interventions and immunomodulation*			
Celecoxib	Chu et al. (2004)	•Rat—collagenase model •Celecoxib (10 or 20 mg/kg) i.p. at 20 min, 6 h, and 24 h after ICH and then daily for 3 or 14 days	•Reduction of PGE2 production, brain edema and inflammation •Better functional recovery
Dexamethasone	Yang et al. (2011)	•Rats—collagenase model •Dexamethasone (15 mg/kg) i.p. injection vs. saline immediately and for 3 days	•Decrease in ICAM-1 and MMP-9 levels •Decrease perihematomal edema ratio
Dimethyl fumarate	Zhao et al. (2015a)	•Autologous blood model •Rat group: 15 mg/kg at 2 h post-ICH i.p. and then orally twice daily for 3 days. •Mice group: administered at 24 h and then day 2 and day 3	•Reduced edema, immune activation, and neurological deficit
Fingolimod	Lu et al. (2014)	•Male mice—collagenase model •Fingolimod 0.5 mg/kg i.p. 30 min after surgery and once a day for 2 days	•Reduced PHE, decreased apoptotic cells in hematoma core. •Improved neurobehavioral recovery •Reduced brain atrophy at 2 weeks
	Schlunk et al. (2016)	•Male mice—collagenase model •Fingolimod 1g/kg i.p. 1 h after ICH induction	•No change in hematoma volume, edema or neurological outcomes and mortality
Siponimod	Bobinger et al. (2019)	•Mice—collagenase model •6 groups: sham, vehicle, low 0.3 mg/kg or high 3 mg/kg dose one (30 min) or multiple administrations (30 min, 24 h, 48 h)	•Declined in lymphocytes •Significant reduction in cerebral edema at 72 h •Longer survival and better neurological outcomes on multiple low doses •No changes to hematoma size
Edaravone	Miao et al. (2020)	•Rat—autologous blood model •Edaravone (3 mg/kg) or vehicle (saline) was administered IV at 2 h and 12 h after ICH and then twice daily for 3 days	•Attenuated brain water content and reduced activation of microglia •Improved motor and behavioral performance
Glibenclamide	Jiang et al. (2017)	•Male rats—autologous blood ICH model •Glibenclamide single loading dose of 10 μg/kg i.p plus subcutaneous infusion at 200 ng/h	•Reduced brain water content in ipsilateral basal ganglia and cortex •Improved motor recovery •Reduced Evans blue leakage 72 h after ICH, reduced the expression of MMP-9
	Kung et al. (2021)	•Male rats—collagenase model •Moderate to severe (40–80 ml) hematoma •Glibenclamide single loading dose of 10 μg/kg i.p at 2 h and i.v. infusion 200 ng/h	•No effect on edema or mortality •No improvement on neurology performance
Memantine	Lee et al. (2006)	•Rat—collagenase model •55 each group memantine (30 min after ICH and OD for 3 days) vs. control	•Neurological improvement for 4 weeks and reduced ICH volume •No difference to water content •Inhibition of apoptosis and neuroinflammation via inhibiting NMDA/t-PA and MMP9 activity
Minocycline	Wasserman and Schlichter (2007)	•Rat—collagenase model •Intraperitoneal Minocycline injection at 6 h, 1 and 2 days	•Reduced: microvessel loss, edema, and extravasation of plasma proteins •Reduced TNF-α and MMP-12 expression •Reduced TNF-α-positive cells and neutrophils
Monascin	Wang et al. (2017)	•Rat—collagenase model •Control vs. 1/5/10 mg/kg/day of monascin	•10 mg/kg/day significantly improved neurological deficits •High dose reduced the volume of hematoma in 1–7 days after ICH •High dose decreased BBB permeability and edema formation in 1–3 days
	Fu et al. (2020)	•Rat—collagenase model •Control vs. 10 mg/kg/day BD by gastric perfusion for 14 days	•Improved long-term outcomes and motor disability •Increased hematoma clearance •Attenuated iron overload and brain atrophy
Therapeutic hypothermia (TH)	John and Colbourne (2016)	•Rat—collagenase model •Delayed (24 h) TH	•No changes to cerebral edema at 7 h or to hematoma size at day •Rapid rewarming worsens cerebral edema •TH protocols significantly reduced average and peak ICP, with benefits persisted after rewarming
Tolvaptan	Tan et al. (2019)	•Rat model •Tolvaptan given at 12, 36, and 60 h after ICH	•Reduced edema •Increased expression of ZO-1 and occludin
*Erythrocytes degradation products*			
Deferoxamine	Nakamura et al. (2004)	•Rat —Autologous blood model •Deferoxamine 100 mg/kg i.p. every 12 h vs. saline •3 groups at 2, 6, and 24 h after ICH	•Reduction of brain edema when treatment began 2 and 6 h after ICH •No difference when it started at 24 h •Improved neurological deficits
*Hematoma volume reduction-neurosurgery*			
Surgery	Wang L. et al. (2015)	•Rabbits—autologous blood •30 each group: control and MIS •ICH evacuated by MIS at 6, 12, 18, 24, and 48 h	•Reduced neurological deficit, more favorable outcome in subgroup 6 to 12 h •Reduced level perihematomal ET-1 •Reduced perihematomal Evans blue and water content. Lowest in 6-h group.

BBB, blood-brain barrier; ET-1, endothelin-1; i.p., intraperitoneal; IV, intravenous; ICAM-1, intercellular adhesion molecule-1; ICH, intracerebral hemorrhage; ICP, intracranial pressure; HTS, hypertonic saline; MIS, minimally invasive surgery; MMP, matrix metalloproteinase; NaCl, sodium chloride; NMDA, N-methyl-D-aspartate receptor; PG, prostaglandin; PGE2; t-PA, tissue plasminogen activator; TH, therapeutic hypothermia; TNF, tumor necrosis factor; ZO-1, zona occluden-1.

### Targeting Na-K-Cl cotransporters

Bumetanide, a Na-K-Cl-cotransporter inhibitor, is used routinely to treat peripheral edema from congestive cardiac failure. In experimental studies, bumetanide has been shown to reduce cytotoxic edema in ischemic stroke, traumatic brain injury, and liver failure (Bautista et al., 2021). However, bumetanide has shown no effect on cytotoxic edema associated with ICH (Wilkinson et al., 2019).

### Targeting aquaporin channels

Tolvaptan is a selective oral Vasopressin V2-receptor antagonist that has been shown to reduce the expression of the AQP4 channel and promote the excretion of excess brain water (Tan et al., 2019). In a preclinical model, administration of Tolvaptan at 12 h, 36 h, and 60 h reduced cerebral edema, prevented degradation of the BBB by downregulating MMP-9, and increased expression of tight junction proteins, occludin and zonula occludens-1 (Tan et al., 2019). Similarly, treatment with Conivaptan has correlated with reduced expression of AQP4 (Corry et al., 2020).

There is preliminary evidence that a novel agent, (nicotinamide)-1,3,4-thiadiazole, TGN-020, and piroxicam, a nonsteroidal anti-inflammatory drug, could also inhibit AQP4 channels (Zhang et al., 2016; Li et al., 2019). More studies are required before translation to clinical settings.

## Interventions for vasogenic edema

### Osmotic agents

Mannitol is one of the most widely used osmotic agents and lowers intracranial pressure by two mechanisms: an immediate effect through plasma expansion, which decreases plasma viscosity, which, in turn, improves regional brain perfusion and oxygenation (Sorani et al., 2008; Diringer et al., 2012; Rickard et al., 2014). Through its osmotic effect, mannitol reduces cerebral edema by drawing water from the brain interstitium into the intravascular compartment (Sorani and Manley, 2008). As a free radical scavenger, mannitol also acts as a neuroprotectant (Bereczki et al., 2007).

Most preclinical studies of mannitol have been in traumatic brain injury, and few studies have tested the effects in acute ICH. One study compared mannitol with two doses of hypertonic saline whereas another tested intravenous infusion of 20% of mannitol every 12 h for 4 doses in total (Qureshi et al., 1999; Schreibman et al., 2018). In both studies, mannitol reduced intracranial pressure and death (Qureshi et al., 1999; Schreibman et al., 2018).

Clinical studies assessing cerebral blood flow have reported varying results from no change with low-dose mannitol (0.9 g/kg) to transient increase using a higher dose (1.5 g/kg) (Misra et al., 2007). A *post-hoc* analysis of a large BP lowering trial showed that mannitol was associated with better outcomes in patients with ICH volume >15 mls (Wang X. et al., 2015; Shah et al., 2018). However, observational data from another study showed no difference in functional outcome at 3 months (Shah et al., 2018).

The precise dose of mannitol for cerebral edema is unknown, but multiple doses can cause severe hypernatremia and high osmolarity, which, in turn, can cause neurological complications, such as osmotic demyelination syndrome (Rickard et al., 2014).

A systematic review assessed the effects of mannitol vs. no mannitol within 24 h of symptom onset (Sun et al., 2018) (Table 2). The conclusion was that treatment should be used only in large hematomas and in patients with cerebral edema or raised intracranial pressure (Sun et al., 2018). However, the studies were mainly retrospective and included a small number of patients, so prospective randomized trials testing the appropriate dose of mannitol, timing, and duration of treatment are warranted (Sun et al., 2018).

**Table 2:** Summary of key clinical trials and meta-analysis of cerebral edema in acute intracerebral hemorrhage.

**Intervention**	**References**	**Method**	**Findings**
*Osmotic agents*			
Mannitol	Sun et al. (2018)	•Meta analysis of 34 studies •*N* = 3,627 •Mannitol vs. control	•No recommendation for use of mannitol in early stages of supratentorial hypertensive intracranial hemorrhage in the absence of clinical signs of intracranial hypertension
*BP-lowering agents*			
Multiple agents	Anderson et al. (2013)	•*N* = 270 •Intensive (<140 mm Hg) vs. guideline-based (<180 mm Hg) BP management	•Attenuated hematoma growth at 72 h •No clear effect on perihematomal edema
	Gong et al. (2017)	•Meta-analysis of 6 studies •*N* = 4,395 •Intensive vs. guideline based	•No significant differences in primary outcomes measures between groups •Higher risk of renal adverse events in intensive group
	Tsivgoulis et al. (2014)	•Meta-analysis of 4 studies •*N* = 3,315 •Intensive vs. guideline-based	•Reduction in absolute hematoma expansion at 24 h
	Moullali et al. (2022)	•Meta-analysis of 50 trials •*N* = 11,494 •Intensive vs. guideline based	•No overall benefit on functional outcome
Nicardipine	Leasure et al. (2019)	•Phase III •*N* = 1,000 •IV nicardipine targeting 3 tiers of SBP: 170–199, 140–169, or 110–139 mm Hg	•Reduction of hematoma expansion and 24-h perihematomal edema ratio in deep ICH •No effect on poor 3-month outcome
*Hematoma volume reduction-neurosurgery*			
Decompressive Craniectomy (DHC)	Yao et al. (2018)	•Meta-analysis: one RCT and 7 observational studies •DHC vs. control •*N* = 286	•DHC significantly reduced mortality rates in those with spontaneous ICH •Not associated with higher rates of postoperative rebreeding or hydrocephalus
Hematoma evacuation	Okuda et al. (2006)	•*N* = 16 •Putaminal hemorrhage •Surgical evacuation vs. conservative treatment	•Hematoma volume reduced by surgery reduces cerebral edema
Early Surgery	Mendelow et al. (2005)	•*N* = 1,033 •Early surgery (within 24 h of randomization) vs. conservative treatment (later evacuation was allowed) •GSC 5 or more; hematoma volume >2 cm •Surgical method: craniotomy, burr hole, endoscopy or stereotaxy	•No differences mortality at 6 months •No statistically significant differences in prognosis based on Rankin scale, Barthel index or Glasgow
	Mendelow et al. (2013)	•*N* =170 •Only traumatic brain injury patients (parenchymal hematomas) •Within 48 of TBI •Hematoma volume > 10 ml •Early surgery (within 12 h of randomization) vs. conservative treatment (later evacuation was allowed)	•Greater survival rate (85% vs. 67%) •6-month outcome (GOS): No significant benefit
	Gregson et al. (2012)	•Individual patient data subgroup meta-analysis •*N* = 2,186 •Surgical vs. conservative management	•Improved outcome (*p* < 0.05) with surgery if performed within 8 h or ICH volume of 20 to 50 ml or GCS 9-12 or age 50–69 years
Burr hole craniectomy	Zuo et al. (2009)	•*N* =176 •Hypertensive basal ganglia hematoma •Gross-total removal vs. sub-total hematoma evacuation	•Significant greater reduction in edema in the complete evacuation group •Higher Barthel index in the complete evacuation group (*p* < 0.05)
MIS plus rtPA	Mould et al. (2013)	•*N* = 79 surgical (hematoma removal using MIS and r-tPA or only surgery) vs. *N* = 39 medical	•Lower edema volume at end of treatment •Correlation between reduction in edema and percentage of ICH removal
MIS	Xia et al. (2018)	•Meta analysis: 5 RCTs and 9 controlled studies •*N* = 2,466 •MIS vs. conventional craniotomy	•MIS was associated with lower rates of rebleeding, better functional recovery •Mortality rates were significantly lower in the MIS group
*Anti-inflammatory interventions*			
Celecoxib	Lee et al. (2013)	•*N* = 44 •within 24 h of ICH •Celecoxib 400 mg BD for 14 days vs. control	•Celecoxib was associated with a smaller expansion of ICH
Dexamethasone	Wintzer et al. (2020)	•Meta-analysis of 7 RCTs •*N* = 490	•No significant benefit or harm of dexamethasone has been established
Fingolimod	Fu et al. (2014)	•*N* = 23 •Fingolimod 0.5 mg orally once a day for 3 days vs. standard care	•Fingolimod reduced relative PHE at day 7 and 14 •No differences in ICH volume
Magnesium	Saver et al. (2013)	•*N* = 1,700 •IV Mg sulfate or placebo within 2 h	•No improvement in functional outcomes at 90 days •Effects on edema volume unknown
Memantine	Bakhshayesh et al. (2014)	•Memantine 10 mg orally daily for a month and then increased to 20 mg daily for 2 months vs. placebo	•Improvement in neurological outcome at 90 days
Minocycline	Fouda et al. (2017)	•*N* = 16 •Within 24 h of onset •400 mg of IV minocycline, followed by 400 mg oral daily for 4 days	•No differences in inflammatory biomarkers (MMP-9, interleukin-6, iron, ferritin, total iron binding capacity), hematoma volume, or perihematomal edema
*Targeting erythrocytes degradation products*			
Deferoxamine (DFO)	Selim et al. (2019)	•Phase II •*N* = 291 •DFO 32 mg/kg/day for 3 consecutive days vs. placebo	•No significant difference on outcome at 90 days •No increased severe adverse events, major disability, or death •No effect on relative PHE growth

BD, ‘bis in die' meaning twice a day; BP, blood pressure; DFO, deferoxamine; DHC, decompressive hemicraniectomy; GCS, Glasgow Coma Scale; GOS, Glasgow Outcome Scale; h, hours; ICH, intracerebral hemorrhage; Mg, magnesium; MIS, minimally invasive surgery; MMP, matrix metalloproteinase; PHE, perihematomal edema; RCT, randomized controlled trial; r-TPA, recombinant tissue plasminogen activator; TBI, traumatic brain injury; TNF, tumor necrosis factor; TXB2, thromboxane B2; vs., versus.

Another osmotic agent, hypertonic saline, has also been assessed. One preclinical study found that intravenous hypertonic saline 3% and 23.4% were equally effective in reducing intracranial pressure (Qureshi et al., 1999). Schreibman et al. combined hypertonic saline with mannitol in moderate to severe ICH and found that there was reduced mortality and swelling at 48 h (Schreibman et al., 2018). In that study, repeated doses of treatment reduced activation of microglia, free-radical formation, and inflammation in the PHE region and the contralateral hemisphere (Schreibman et al., 2018). Despite this evidence, there are few data on the clinical effects of hypertonic saline, and no randomized trials have been performed (Wagner et al., 2011). Hypertonic saline can cause electrolyte imbalance, volume overload, coagulopathy, and rebound increase in intracranial pressure, so patients have to be carefully monitored (Diringer and Zazulia, 2004; Rickard et al., 2014).

Preclinical studies have shown that the combination of furosemide and mannitol can be more effective in reducing intracranial pressure and reversing the osmotic gradient in ICH than mannitol alone (Thenuwara et al., 2002). Similar results have been observed with furosemide and hypertonic saline compared to hypertonic saline (Mayzler et al., 2006). There is a risk of electrolyte disturbance and neurological complications, but it is thought to be not higher with two osmotic agents compared to one (Pollay et al., 1983; Thenuwara et al., 2002; Diringer and Zazulia, 2004). This might mean that combining two agents for cerebral edema is synergistic but needs to be evaluated further.

### Thrombin inhibition

There is evidence that suggests that inhibition of thrombin can reduce vasogenic edema after acute ICH (Xi et al., 2003). Treatment with a thrombin inhibitor such as argatroban might seem counterintuitive as thrombin is essential for coagulation. However, thrombin which is bound to fibrin for the first few days after stroke is released afterwards leading to delayed cerebral edema (Kitaoka et al., 2002). So, starting treatment after a hematoma has stabilized may be useful. In a small study, intravenous argatroban was tested 24 h after ictus in a rat ICH model and the results showed that treatment was associated with a reduction in perihematomal edema (Nagatsuna et al., 2005). In addition, thrombin was also found to suppress the infiltration of neutrophils and macrophages (Nagatsuna et al., 2005). This suggests that argatroban might be a potential therapeutic option for ICH edema but studies are required to assess the appropriate dose, safety, and efficacy (Bath, 2012).

### Anti-inflammatory agents

During the inflammatory phase of edema, the selective depletion of neutrophils has been shown to reduce BBB permeability, MMP-9 expression, and axonal damage and improve outcomes in experimental models (Moxon-Emre and Schilchter, 2011; Sansing et al., 2011b). Similarly, inhibiting MMP or treatment with toxic free radical scavengers or a TNF-α antibody could reduce perihematomal edema, but prospective clinical studies are awaited (Imai et al., 2019; Lattanzi et al., 2020).

Minocycline is a second-generation tetracycline antibiotic that is able to enter the brain through the BBB following a stroke (Wasserman and Schlichter, 2007). Minocycline inhibits the activation of microglia, and in one preclinical study, the treatment reduced BBB permeability, prevented extravasation of plasma proteins, and showed antioxidant and antiapoptotic properties (Wasserman and Schlichter, 2007; Chang et al., 2017). Studies have also shown that minocycline reduces iron overload and associated brain damage (Zhao et al., 2011; Chang et al., 2017; Cao et al., 2018). In a clinical trial, treatment with minocycline was safe and tended to reduce MMP-9 after 3–5 days (Fouda et al., 2017). However, the study was unable to assess the effect on edema volume or functional outcome (Fouda et al., 2017).

Tranexamic acid is a well-known antifibrinolytic agent that has been shown to be effective in reducing bleeding after trauma and postpartum hemorrhage (Shakur et al., 2010; Trial Collaborators WOMAN, 2017). It has been tested in acute ICH, and in a large trial, the Tranexamic Acid for Hyperacute Primary Intracerebral Hemorrhage (TICH-2) trial, treatment within 8 h was associated with reduced hematoma expansion and early death, but there was no difference in functional outcome at day 90 (Sprigg et al., 2018). Due to the mechanism of action, there is a risk of brain ischemia, but a substudy of TICH-2 reported no difference in patients who received tranexamic acid compared to those who received placebo (Josephson et al., 2022). Experimental studies suggest that tranexamic acid also has anti-inflammatory effects by decreasing TNF-α, IL-1β, and M1 macrophages and increasing the levels of the M2 macrophage phenotype (Barrett et al., 2019; Yoshizaki et al., 2019). Whether these putative effects translate into the clinical setting needs to be assessed.

Celecoxib, a well-known cyclo-oxygenase-2 inhibitor, has been tested in acute ICH and shown to reduce inflammation and edema associated with prostaglandin E2 (Chu et al., 2004). In a pilot, clinical trial, patients randomized to 14 days of celecoxib (400mg twice daily) had a significant reduction in edema volume compared to controls (Lee et al., 2013). However, the time from ictus to the first CT scan was different between the two groups, which could have impacted the results (Lee et al., 2013).

The rationale for using steroids such as dexamethasone is to minimize the mass effect of edema and inflammation (Lee et al., 2015). Dexamethasone could promote recovery after ICH because it decreases apoptotic cell death and inhibits the expression of MMP-9 (Yang et al., 2011; Lee et al., 2015). Similar to celecoxib, preclinical studies of dexamethasone have shown positive effects (Yang et al., 2011; Lee et al., 2015), but this has not been translated to clinical trials. A meta-analysis including 248 patients found that there was no evidence of a benefit from dexamethasone, so its use for routine treatment for ICH edema is not recommended (Wintzer et al., 2020).

Experimental studies have shown that selective deactivation of the complement cascade could inhibit brain inflammation (Xi et al., 2002; Gong et al., 2005). For example, N-acetyl heparin, which is a derivative of heparin, inhibits complement without any effect on coagulation (Gong et al., 2005). It is also known to inhibit thrombin-induced complement activation (Gong et al., 2005). This proof of concept was shown in one small preclinical study, with a reduction in brain edema at 24 and 72 h. Work on this has since been reproduced and extended to effects including the reduction of brain atrophy at 1 month after ICH (Gong et al., 2005).

There is emerging evidence that peroxisome proliferator-activated receptor–gamma (PPAR-γ), a transcription factor, is important for modulating inflammation and phagocytosis. Therefore, testing PPAR-γ agonists such as pioglitazone and rosiglitazone could lead to reduction of hematoma and enhance recovery (Zhao et al., 2006, 2007a,b). These agents have been tested in preclinical studies, but there is little clinical data for ICH (Zhao et al., 2007a; Wu et al., 2015). Preliminary work indicates that through specific inhibition of TLR-4 on macrophages, outcomes could improve, but more research is needed (Wu J. et al., 2017).

Recently, the recombinant human IL-1 receptor antagonist Anakinra has been shown to reduce inflammation in preclinical models of ICH (Smith et al., 2018). Based on these results, two phase 2 studies are testing its effects within 8 h of symptom onset (NCT04834388; NCT03737344).

## Immune modulation

Fingolimod, a sphingosine 1-phosphate receptor analog (S1 P-R), is able to cross the BBB and act directly on neurons and glial cells (Fu et al., 2014). In preclinical studies using collagenase models of ICH, treatment with fingolimod was associated with less cerebral edema and lymphocytic proliferation and decreased apoptosis (La Mantia et al., 2016). Other studies have shown that fingolimod is neuroprotective and improves behavioral outcomes (Lu et al., 2014). A phase 2 clinical study has shown that treatment was associated with reduced perihematomal edema volume (Fu et al., 2014), and this has led to testing a single-dose 0.5 mg fingolimod as potential treatment within 24 h of ictus (NCT04088630) (Wolfe, 2022).

Siponimod is a more selective S1 P-R analog but has a shorter half-life compared to fingolimod. Experimental studies of siponimod showed that it reduced cerebral edema and improved neurological recovery and survival (Bobinger et al., 2019, 2020). However, a phase 2 randomized clinical trial (NCT03338998) was terminated due to a lack of efficacy (NCT03338998, 2022).

## Putative neuroprotectants

Edaravone, which is a novel synthetic molecule with scavenging properties, is an attractive neuroprotectant. In one preclinical study, edaravone reduced brain water content, suppressed inflammation by blocking the release of NLRP3 [nucleotide binding oligomerization domain (NOD), leucine-rich repeat (LRR), and pyrin domain–containing protein 3], and improved motor and cognitive outcomes (Miao et al., 2020). Little is known of the clinical effects, and so a small trial (NCT04714177) has begun testing the effects of edaravone in hypertensive ICH patients within 48 h of onset (NCT04714177, 2021).

One study tested disufenton, a free-radical trapping agent, in patients with ICH, but there was no benefit (Lyden et al., 2007). The reasons could be reflected in the insufficient permeability of disufenton to enter through the BBB or that a single agent is not enough to neutralize the large numbers of free radicals that are generated (Lyden et al., 2007). Other agents such as pyrroloquinoline quinone and sulforaphane, which activate a protein nuclear factor (erythroid2-related factor 2), have been tested for their antioxidant properties and whether they enhance brain recovery in experimental stroke (Ma et al., 2015; Bautista et al., 2021). The clinical effects are yet to be established.

Given the inability of free radical scavengers to affect the neurological outcomes in patients with acute ischemic stroke (Serebruany, 2006; Shuaib et al., 2007) and the prominent role of pro-oxidant enzyme Nicotinamide adenine dinucleotide phosphate (NADPH) oxidase in cerebrovascular ROS generation (Serebruany, 2006; Shuaib et al., 2007; Allen and Bayraktutan, 2009), it is conceivable that the inhibition of this enzyme system or its functional subunits (p22-phox or gp91-phox) may prove efficacious. Indeed, suppression of NADPH oxidase has been shown to negate its effects in acute ischemia, hyperglycemia, or TNF-? on an *in vitro* model of human BBB through regulation of apoptosis, MMP-2/9 and the protein kinase C pathway (Abdullah and Bayraktutan, 2014; Rakkar et al., 2014; Shao and Bayraktutan, 2014).

Magnesium is appealing as a neuroprotectant because of its membrane-stabilizing effects, and it has been shown to increase cerebral blood flow, enhance recovery, and reduce damage from toxic free radicals (Avgerinos et al., 2019). Studies have shown that it is feasible to administer intravenous magnesium in acute stroke (Saver et al., 2004), but in one phase 3 trial, treatment within 2 h had no effect on neurological recovery or functional outcome (Saver et al., 2013). Potential explanations for these results include the small number of patients with ICH and the timing could have been too early for treatment to have any effect on edema (Saver et al., 2013).

Inhibition of excess accumulation of glutamate in acute ICH with memantine, a fast-acting, noncompetitive NMDA channel antagonist, has been tested (Lee et al., 2006). One preclinical trial of memantine reported an approximate 50% reduction in ICH volume coupled with the inhibition of MMP-9 following treatment for 14 days (Lee et al., 2006). In another study, 64 patients were allocated to either memantine (10 mg daily for a month and then increased to 20 mg daily for 2 months) or placebo within 24 h of ictus (Bakshayesh-Ebhbali and Hajinnori, 2015). At 3 months, patients treated with memantine reported better motor function compared to those receiving the placebo (Bakshayesh-Ebhbali and Hajinnori, 2015). Data also suggest that memantine may improve cognition and aphasia (Berthier et al., 2009), but this needs to be tested further.

Statins have been shown to be neuroprotective by modulating cellular pathways and controlling leukocyte proliferation, adhesion, migration, and angiogenesis (Tapia-Perez et al., 2013). Preclinical studies have shown that these effects might translate to clinical recovery through hematoma resolution and reduced cerebral edema (Jung et al., 2004; Yang et al., 2013; Chen et al., 2019). Based on these results and retrospective data showing lower mortality, the Statin for Neuroprotection in Spontaneous Intracerebral Hemorrhage trial (STATIC) is now testing the effect of Atorvastatin 20 mg in ICH-associated cerebral edema (Chen et al., 2019) (Appendix).

Other neuroprotective agents include monascin, which is a natural agonist of the PPAR. In preclinical models, monascin has been shown to reduce ICH volume and reduce cerebral edema and BBB permeability at higher doses (Wang et al., 2017; Fu et al., 2020). Further testing of this agent is awaited.

## Hypothermia

Therapeutic hypothermia and fever prevention have the potential to be therapeutic options for ICH edema (Loach and Bayraktutan, 2016; Greer et al., 2021). Preclinical studies have shown promise, and a meta-analysis concluded that hypothermia reduced edema-associated BBB disruption through the suppression of excitotoxicity, inflammation, oxidative stress, and apoptosis (Staykov et al., 2010; John and Colbourne, 2016; Loach and Bayraktutan, 2016; Melmed and Lyden, 2017). Furthermore, therapeutic hypothermia has been shown to augment the BBB-reparative capacity of therapeutic agents targeting protein kinase C-β or the NADPH oxidase enzyme system (Kadir et al., 2021). One prospective clinical study tested endovascular mild hypothermia (35 degrees celcius for 10 days) in patients with moderate volume ICH (Staykov et al., 2013). The study reported that the volume of edema remained stable throughout treatment, and, interestingly, there was no rebound increase after patients had been rewarmed to normal temperature (Staykov et al., 2013). Therapeutic hypothermia is associated with complications such as pneumonia, coagulopathy, and bradycardia, which appear to be directly related to the depth and duration of cooling (Zhao et al., 2007a; John and Colbourne, 2016). Therefore, optimizing cooling protocols might prevent such complications, but this needs to be assessed in future studies (Staykov et al., 2010; Loach and Bayraktutan, 2017).

## Targeting erythrolysis and products of degradation

Deferoxamine has been shown to be neuroprotective and improve outcomes in experimental ICH (Staykov et al., 2010; Selim et al., 2011). As a chelator, it reduces the amount of free iron that is available to cause cerebral edema and neurotoxicity. Deferoxamine has also been shown to have other effects: it has antioxidant properties that directly reduce neuronal damage; downregulate cytokines, particularly TNF-α; and activate the enzyme cyclooxygenase, thereby reducing brain inflammation and inhibiting toxicity caused by hemoglobin (Nakamura et al., 2004; Okauchi et al., 2009). A meta-analysis analyzing the effect of deferoxamine in preclinical models of ICH found that treatment reduced brain water by 85% and improved neurobehavioral outcomes (Okauchi et al., 2009). In addition, this analysis reported that treatment was effective if started within the first few hours of ictus (Okauchi et al., 2009).

Based on these results, a phase II clinical trial tested deferoxamine at a dose of 32 mg/kg/day (Selim et al., 2019). Participants were enrolled within 24 h of ICH, and treatment was given for 3 consecutive days (Selim et al., 2019). The results showed that deferoxamine was safe, but there was no difference in the primary outcome of death or dependency at day 90 between deferoxamine compared to placebo (34% vs. 33%) (Selim et al., 2019). A *post-hoc* analysis suggested a treatment effect at 6 months, indicating that the benefits with deferoxamine might be observed after a longer period but there was no effect on cognition, mood, or PHE (Wei et al., 2021). The results do not support testing of deferoxamine on its own in a future study, but combining it with another intervention could be an option (Wei et al., 2021).

## Endothelial progenitor cells

Cell therapy with endothelial progenitor cells (EPCs), bone marrow–derived circulating stem cells, may induce therapeutic angiogenesis and potentially improve outcomes after ICH. Translational and clinical studies indicate that EPCs may potentiate endothelial cell regeneration, a prerequisite for effective BBB repair and neovascularization (Bayraktutan, 2019; Kadir et al., 2022a,b). Indeed, EPC levels are associated with good neurological outcomes and reduced hematoma volume in ICH, implying the potential as a novel therapeutic option for patients (Pías-Peleteiro et al., 2016).

## Surgery

Surgery for ICH has theoretical benefits for the management of edema: it reduces mass effect, lowers intracranial pressure (ICP), and removes inflammatory and neurotoxic blood products. Open craniotomy is the most studied approach, but two well-powered randomized controlled trials did not show a benefit of surgery compared to medical management alone (Mendelow et al., 2005, 2013). A pooled analysis of the two trials with results of previous studies suggested that early surgery irrespective of the technique was associated with a survival benefit in a subgroup of patients (e.g., those who deteriorate neurologically from hematoma expansion or superficial, lobar ICH with no intraventricular extension) (Gregson et al., 2012). It is important to highlight that the two trials were based on clinical equipoise, i.e. patients thought to benefit from surgery at the outset were excluded (Mendelow et al., 2005, 2013). Moreover, the protocols allowed cross-over of patients to craniotomy if the patient deteriorated (Mendelow et al., 2005, 2013). As a result, the actual benefit of delayed surgery in ICH is unclear and could have been underestimated.

Preclinical studies have reported that early minimally invasive surgery is associated with reduced extracellular glutamate, perihematomal endothelin-1 levels, BBB permeability, and brain water content (Wu et al., 2013; Wang L. et al., 2015). In a small study, patients with putaminal hematomas who underwent surgery had significantly reduced cerebral edema on follow-up scans (Okuda et al., 2006). However, a decrease in edema has been shown to depend on the amount of hematoma that is evacuated (Horowitz et al., 2021). Another analysis compared the effect of hematoma drainage by burr hole craniectomy on severity of cerebral edema (Zuo et al., 2009). The results showed that at 3 weeks, surgery significantly reduced levels of TNF-α and products released by the coagulation cascade (Zuo et al., 2009). Although hematoma evacuation is suggested to reduce perihematomal edema, it is unclear whether it affects outcomes. One study analyzed the relationships between the ratio of residual hematoma volume to edema, inflammation, and prognosis (Zuo et al., 2009). At 1 year, patients who had complete evacuation of hematoma had better functional outcomes (Zuo et al., 2009). A *post-hoc* analysis of a phase 3 trial reported a 35% reduction in perihematomal edema, but there was no improvement in functional outcomes (Hanley et al., 2019).

One factor that can increase edema is intraventricular hemorrhage (IVH) because there is an imbalance in the absorption of cerebrospinal fluid. This often leads to acute hydrocephalus, which, in turn, worsens edema by increasing ICP, reduces brain perfusion, and induces ventricular wall stretch, white matter injury, and inflammation (Holste et al., 2022). Therefore, placement of an external ventricular drain (EVD) could reduce these effects. Observational data suggest that placement of EVD in IVH is associated with reduced mortality, but the effect on long-term outcomes is unclear (Lovasik et al., 2016). It is also important to highlight that placement of EVD has its challenges including blockage, malpositioning, overdrainage, the need for prolonged drainage, and infection (Jamjoom et al., 2018). To date, there have been no randomized trials of EVD in ICH, therefore research into the potential benefits is needed.

Decompressive hemicraniectomy (DHC) refers to the removal of a window of bone with wide durotomy or dural expansion to allow for normalization of ICP and mass effect. DHC is routinely performed in malignant middle cerebral artery (MCA) infarction, but it is less established for the treatment of spontaneous ICH (Yao et al., 2018). The effect of DHC for edema after ICH is unclear, and the indication must be balanced against the complications, including hematoma expansion (~25% of cases) and an additional procedure, that is, cranioplasty (Gopalakrishnan et al., 2018). One randomized trial is evaluating whether DHC with the best medical treatment improves outcomes vs. best medical treatment alone (NCT02258919, 2023) (Appendix).

## Conclusion and future perspectives

As of yet, there is no established treatment for prevention or reducing PHE, but information on which ones show promise and those that may not be effective is emerging. Some drugs used in other medical conditions have properties that could be useful in treating cerebral edema and offer the possibility of being repurposed.

Lowering BP is recommended after acute ICH, but its routine use and for secondary prevention means that specific testing for effects on PHE might necessitate a comparison of intensity (standard vs. guideline) or agent. Osmotic agents have biologically plausible mechanisms that might be effective for treating PHE, but more work is needed on the optimal dose, treatment duration, safety, and efficacy. Because hematoma volume is strongly linked with cerebral edema, surgery may still have a role, but more research is needed on the timing and technique to identify which patients benefit.

The involvement of AQP4 channels in the pathogenesis of cytotoxic edema is clear, but more work is needed to develop effective agents. The SuR1-TRPM4 channel seems to be a key target, and glyburide as a potential inhibitor is promising. One concern is that treatment might increase the risk of hypoglycemia, although this was not observed in a small phase 2 trial testing the effects of glyburide in ischemic stroke (Sheth et al., 2016).

Agents that are neuroprotective and remove toxic free radicals are promising as are treatments that attenuate inflammation and infiltration of leucocytes into the perihematomal region after ICH. With careful selection, it is possible that some of these could translate from preclinical to clinical studies. This review also highlights that a better understanding of the role of inflammation and the immune system after stroke is needed as the potential for modulation could have profound implications for clinical management.

As highlighted, the mechanisms of PHE are complex with the magnitude time-dependent, and this should be taken into consideration when designing future clinical studies. For example, a trial could test reducing hemorrhage expansion first and, when the bleeding has stopped, then assess evacuation followed by an anti-inflammatory or neuroprotective agent. In the acute phase, it may be that osmotic agents complement BP lowering, hemostatic therapy, or surgery for ICH. Such an innovation would warrant streamlining of trial protocols, careful patient selection, and incorporation of specific outcomes to test response and recovery. This work will need close collaboration between clinicians, researchers, pharmaceutical companies, and industry.

The present review also highlights that there is variation in the measurement of PHE and the modality of brain imaging, therefore more research is needed to identify a standard to assess change or severity. Evidence suggests that brain repair and recovery can continue for months after ICH (Selim et al., 2019), therefore testing the long-term effects of potential treatments for cerebral edema is warranted.

## Author contributions

KK: Conceptualization, Writing—original draft, Data curation, Methodology, Resources. PC: Writing—original draft, Data curation, Resources. TN: Writing—review and editing. CT: Data curation, Writing—original draft, Resources. SC: Data curation, Writing—original draft, Resources. JA: Writing—review and editing. ZL: Writing—original draft, Resources. MH: Writing—original draft. MK: Writing—review and editing. TE: Writing—review and editing. CR: Writing—review and editing. MM: Writing—review and editing. JD: Writing—review and editing. UB: Writing—original draft. DW: Writing—review and editing. NS: Writing—review and editing. PB: Writing—review and editing.

## Appendix

**Table A1:** Table of ongoing studies of cerebral edema in intracerebral hemorrhage.

**Study/trial acronym; NCT Identifier**	**Intervention**
ACTION NCT04834388	Anakinra 500 mg intravenous loading dose, followed by infusion 2 mg/kg/h over 3 days vs. anakinra 100 mg subcutaneous injection of 100 mg twice daily for 3 days vs. standard care
NCT04890379	Dimethyl fumarate 240 mg orally twice daily for 3 days vs. placebo
ED-ICH NCT04714177	Edaravone dexborneol 37.5 mg injection BD for 14 days vs. placebo
ICH ADAPT NCT02281838	Target systolic blood pressure <140 mm Hg vs. <180 mm Hg within 6 h of stroke onset
FITCH NCT04088630	Single dose of 0.5 mg oral fingolimod within 24 h of symptom onset vs. placebo
STATIC NCT04857632	Atorvastatin 20 mg daily for 7 days vs. standard care
DIST NCT03608423	Minimally invasive endoscopy-guided surgery or hematoma aspiration, additional to standard medical treatment vs. best medical care
INVEST NCT02654015	Minimally Invasive Endoscopic Surgical Treatment With Apollo/Artemis in Patients With Brain Hemorrhage
MIND NCT03342664	A Prospective, Multi-Centre Study of Artemis a Minimally Invasive Neuro Evacuation Device, in the Removal of Intracerebral Hemorrhage
EVACUATE NCT04434807	Ultra-early, minimally invasive intracerebral hemorrhage evacuation vs. standard treatment
SWITCH NCT02258919	Decompressive Hemicraniectomy plus best medical care vs. best medical care alone
